# Online Hierarchical Sparse Representation of Multifeature for Robust Object Tracking

**DOI:** 10.1155/2016/5894639

**Published:** 2016-08-18

**Authors:** Honghong Yang, Shiru Qu

**Affiliations:** Department of Automation, Northwestern Polytechnical University, Xi'an 710072, China

## Abstract

Object tracking based on sparse representation has given promising tracking results in recent years. However, the trackers under the framework of sparse representation always overemphasize the sparse representation and ignore the correlation of visual information. In addition, the sparse coding methods only encode the local region independently and ignore the spatial neighborhood information of the image. In this paper, we propose a robust tracking algorithm. Firstly, multiple complementary features are used to describe the object appearance; the appearance model of the tracked target is modeled by instantaneous and stable appearance features simultaneously. A two-stage sparse-coded method which takes the spatial neighborhood information of the image patch and the computation burden into consideration is used to compute the reconstructed object appearance. Then, the reliability of each tracker is measured by the tracking likelihood function of transient and reconstructed appearance models. Finally, the most reliable tracker is obtained by a well established particle filter framework; the training set and the template library are incrementally updated based on the current tracking results. Experiment results on different challenging video sequences show that the proposed algorithm performs well with superior tracking accuracy and robustness.

## 1. Introduction

The task of visual tracking is to find the interested object and track it. It is an important research in computer vision due to its widespread applications in traffic monitoring, vehicle navigation, and visual surveillance. Robust object tracking in dynamic environment is still a challenging problem. This is mainly because the factors such as occlusion, pose variation, illumination change, and clutter background cause large appearance change [1, 2].

A robust appearance model is important for dealing with occlusions or other interferences in the tracking process. A target object is represented by its visual information like color, edge, or texture features extracted from the target region. However, there are numerous trackers only that rely on single feature to build target appearance, ignore the complementary representation of different features, usually lack of robustness, and are sensitive to interferences in dynamic environment [3]. For example, Ross et al. [4] use the intensity feature to represent the appearance model of the target object and integrate incremental learning to obtain a low-dimensional subspace representation. Babenko et al. [5] propose Multiple Instance Learning (MIL), which employs the Haar-like feature to build the discriminative appearance model for robust tracking. Mei and Ling [6] introduce a *l*_1_ minimization robust visual tracking method, it uses the intensity feature to represent the target appearance, and the target appearance is represented by sparse linear combination of the appearance template and trivial template in template space. However, the single feature ignores the complementary characteristic of different visual information; it is insufficient to describe the drastic changes of target appearance in complicated environment. Therefore, the representation ability will decline when there are occlusions or other interferences in complex background [3]. As a result, numerous trackers are proposed to represent the object by fusing the multiple features to describe the target object and build the object appearance model, which can better describe the appearance changes and is beneficial to improve the robustness of trackers in dynamic environments [7–11]. However, how to effectively use and integrate multiple features for robust tracking should be tackled urgently.

Numerous trackers based on sparse representation have been proposed in recent years [12–17]. Mei et al. [6, 17] proposed a *l*_1_ minimization robust tracking method that regards the tracking as a sparse approximation problem. Zhang et al. [14] proposed a low-rank sparse representation tracking method. Liu et al. [15] developed a robust tracking algorithm using a local sparse appearance model to balance the requirements of stability and flexibility in the process of tracking. These trackers are all solved as the sparse approximation problem by *ℓ*_1_ regularized least squares method and show promising results against many existing trackers. However, this sparse coding based on *ℓ*_1_ minimization provides very sparse representation but ignores the importance of collaboration representation and the correlation of visual information; it is vulnerable to interferences and the *ℓ*_1_ minimization is very time-consuming. In [16], Zhang et al. emphasized the role of collaborative representation with *ℓ*_2_ regularized least squares, which shows that *ℓ*_2__RLS is beneficial for reducing the computation burden. Shi et al. [18] also demonstrated that *ℓ*_2__RLS is more accurate, robust, and faster. In [19], Yu et al. demonstrated that traditional sparse coding methods ignore the spatial neighborhood structure of the image because they only encode the local patches independently; then they proposed an efficient discriminative image representation method by using a two-layer sparse coding scheme at the pixel level.

**Figure 1:**
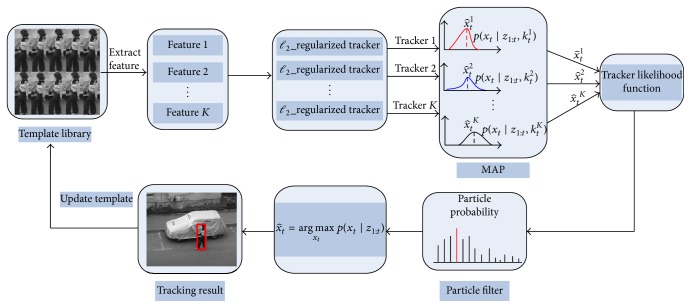
The framework of the proposed tracking algorithm.

Inspired by the challenges mentioned above, this paper proposes an object tracking algorithm that combines the multiple visual features with hierarchical sparse coding to realize the tracking. As shown in Figure 1, a multiple complementary feature representation [20] is used to robustly represent the object; the target appearance is modeled by exploiting a two-stage sparse-coded method, which is based on *ℓ*_2_ regularized least squares to solve the sparse approximation problem. Then, each tracker is based on the different features to estimate the object state and build the multiple observation models. The corresponding reliability of each tracker is computed by the tracking likelihood function of instantaneous and reconstructed appearance models that take the transient and stable appearance changes into consideration. Finally, the most reliable tracker is obtained by a well established particle filter framework; the training set and template library are incrementally updated based on the current tracking result.

The main contributions of the proposed tracking algorithm are as follows: (1) we construct the target appearance by taking account of instantaneous and stable appearance features; then the transient appearance model and reconstructed object appearance model are built independently. (2) A two-stage sparse-coded method is employed to obtain the reconstructed coefficient vector used to construct the reconstructed appearance model. The two-stage sparse-coded method takes the temporal correlation between target templates and spatial neighborhood structure of the image patches into consideration and solves the sparse approximation problem by *ℓ*_2__RLS. This is beneficial for reducing the computational burden and improving the tracking performance. (3) To better describe the object appearance changes, the reliability of each tracker is measured by the tracking likelihood function of instantaneous and reconstructed appearance model that take transient and stable appearance changes into consideration. Experimental results on challenging sequences show that the proposed method performs well compared to state-of-the-art methods.

## 2. The Proposed Tracking Algorithm

### 2.1. Particle Filter Tracking Framework

Given the object observations in previous *t*th frame *z*_1:*t*_ = {*z*_1_, *z*_2_,…, *z*_*t*_}, the object state in *t*th frame is defined as *x*_*t*_ = [*x*_*t*_, *y*_*t*_, *s*_*t*_, *θ*_*t*_, *ε*_*t*_, *ϕ*_*t*_]^*T*^, where *x*_*t*_, *y*_*t*_ denote the coordinates, *s*_*t*_, *θ*_*t*_ are the scale and aspect, and *ε*_*t*_, *ϕ*_*t*_ are the rotation angle and skew. In order to robustly represent the object, we use multifeatures to build observation models of multiple trackers, let *k*_*t*_ ∈ {1,…, *K*} denote the index of *K* trackers from *K* features, then the *i*th tracker's index is *k*_*t*_^*i*^.

The *i*th tracker's posterior distribution of the state *x*_*t*_ is

(1) $$\begin{array}{c} p\left(\;x_{t}\;|\;z_{1:t},k_{t}^{i}\;\right)=p\left(\;z_{t}\;|\;x_{t},k_{t}^{i}\;\right)p\left(\;x_{t}\;|\;z_{1:t-1},k_{t}^{i}\;\right), \end{array}$$

where *p*(*z*_*t*_∣*x*_*t*_, *k*_*t*_^*i*^) is the observation model and *p*(*x*_*t*_∣*z*_1:*t*−1_, *k*_*t*_^*i*^) is the predicted distribution of *i*th tracker:

(2) pxt ∣ z1:t−1,kti=∫pxt ∣ xt−1,ktipxt−1 ∣ z1:t−1,ktidxt−1,

(3) pxt−1 ∣ z1:t−1,kti=∑j=1Kpxt−1 ∣ z1:t−1,kt−1jPkt−1j ∣ kti,z1:t−1,

where *p*(*x*_*t*_∣*x*_*t*−1_, *k*_*t*_^*i*^) is the motion model of *i*th tracker between the *t*th and (*t* − 1)th frame, which is restricted to Gaussian distribution *N*(*x*_*t*_∣*x*_*t*−1_ *σ*). *p*(*x*_*t*−1_∣*z*_1:*t*−1_, *k*_*t*_^*i*^) denotes the prior distribution up to frame *t* − 1 and *P*{*k*_*t*_^*i*^∣*z*_1:*t*_} is the probability of the *i*th tracker.

The crossover probability of *i*th tracker for multiple features is

(4) Pkt−1j ∣ kti,z1:t−1=Pkti ∣ kt−1j,z1:t−1Pkt−1j ∣ z1:t−1∑l=1KPkti ∣ kt−1l,z1:t−1Pkt−1l ∣ z1:t−1.

In addition, the *i*th tracker probability *P*{·} satisfies

(5) ∑iPkti ∣ z1:t=1,∑jPkt−1j ∣ kti,z1:t−1=1.

Then, we sparsely represent the candidate sample *z*_*t*_ with state *x*_*t*_^*i*^ from the template library *f*_*t*_^*k*^; the likelihood of the observation model is

(6) $$\begin{array}{c} p\left(\;z_{t}\;|\;x_{t},k_{t}^{i}\;\right)=\exp\left(\;-\varepsilon_{i}\;\right), \end{array}$$

where *ε*_*i*_ = min⁡‖*fα* − *z*_*t*_‖ is the sparse reconstruction error of candidate sample *z*_*t*_ and *α* is the sparse coefficients.

Therefore, the tracking result ${\hat{x}}_{t}$ at the *t*th frame is the most reliable tracker with the highest tracker probability:

(7) x^t=arg⁡maxxt⁡ pxt ∣ z1:t,k^t,k^t=arg⁡maxkti⁡ Pkti ∣ z1:t,i=1,…,K.

### 2.2. Multiple Features Representation for Object Appearance

The different features have complementary characteristics to cope with appearance changes, such that the HOG features are robust to pose variations [21], Haar-like features can effectively deal with occlusions [22] as the single appearance model is insufficient to represent the target in a complicated environment. Therefore, we exploit different types of the features to build the multiple appearance models to represent the object robustly. The multiple features with complementary characteristics are used to handle various appearance changes, which is beneficial for tracking the target object robustly.

In the proposed method, we use three trackers based on HOG, Haar-like feature, and intensity feature to represent the object appearance, which can effectively deal with occlusions, illumination changes, and pose variations. For the *t*th frame, we extract the multiple features to form feature sets as *f*_*t*_^*k*^ ∈ *ℝ* ^*m*_*k*_^, where *k* is the index of the feature and *m*_*k*_ is the dimension of the *k*th feature. Normalize the feature sets *f*_*t*_^*k*^ ∈ *ℝ* ^*m*_*k*_×*n*^ to form the target template *f*_*t*_^*k*^ ∈ *ℝ* ^*d*_*k*_^ and *d*_*k*_ denotes the dimension of *k*th multiple features.

### 2.3. Object Representation by Hierarchical Sparse Coding

In the proposed method, we use the transient and stable features to describe the abrupt and stable object appearance changes. The stable features are sparsely represented by the current template with hierarchical sparse coding. Then, the reliability of each tracker is measured by the tracking likelihood function of instantaneous and reconstructed appearance models.

The transient features up to *t*th frame is *f*_*I*,*t*_^*k*^ = [*f*_*I*,*t*−*l*_^*k*^,…, *f*_*I*,*t*−1_^*k*^]. Then the transient appearance model ${\overset{-}{f}}_{I,t}^{k}$ is achieved by averaging the recent *L* appearance features as

(8) $$\begin{array}{c} {\overset{-}{f}}_{I,t}^{k}=\frac{1}{L}\overset{L}{\underset{l=1}{\sum }}f_{I,t-l}^{k}. \end{array}$$

The stable object appearance *z*_*t*_^*i*,*k*^ is represented by sparse coding the stable features *f*_*R*,*t*_^*k*^ as

(9) $$\begin{array}{c} z_{t}^{i,k}\approx f_{R,t}^{k}\alpha_{t}^{i,k}+\varepsilon^{i,k}=f_{1,t}^{k}\alpha_{1,t}^{i,k}+f_{2,t}^{k}\alpha_{2,t}^{i,k}+\cdots +f_{r,t}^{k}\alpha_{r,t}^{i,k}. \end{array}$$

Because the tracking algorithm based on sparse representation is to find samples with minimal reconstruction errors from the templates library, a target can be reconstructed from several templates [23]. Therefore, there are only some features having the discriminative capability to separate the target from its background. In order to achieve the goals that discriminatively separate the target from its background and minimal reconstruction errors from its template library, we utilize the hierarchical sparse coding to minimize reconstruction errors and maximize the discriminative capability of features. In addition, we use *ℓ*_2__RLS to solve the sparse approximation problem, which is beneficial for reducing the computation burden.

Let $f_{1}=\left[\begin{array}{cc} f_{R,t}^{k} & I^{k} \end{array}\right]$, $\alpha_{1}=\left[\begin{array}{c} \alpha_{t}^{i,k}\\ \beta_{t}^{i,k} \end{array}\right]$, where **α**_*t*_^*i*,*k*^ = [*α*_1,*t*_^*i*,*k*^,…, *α*_*r*,*t*_^*i*,*k*^]^*T*^ ∈ *ℝ* ^*r*^ is the sparse coefficient vector, **β**_*t*_^*i*,*k*^ = [*β*_1,*t*_^*i*,*k*^,…, *β*_*d*^*k*^,*t*_^*i*,*k*^]^*T*^ ∈ *ℝ* ^*d*^*k*^^ is the noise coefficient vector, and **I** ^*k*^ ∈ *ℝ* ^*d*^*k*^×*d*^*k*^^ is an identity matrix. The candidate sample *z*_*t*_^*i*,*k*^ is sparsely represented by linear combination of the features *f*_*t*_^*i*,*k*^ as

(10) $$\begin{array}{c} z_{t}^{i,k}=f_{R,t}^{k}\alpha_{t}^{i,k}+\varepsilon^{i,k}=\left[\begin{array}{cc} f_{R,t}^{k} & I \end{array}\right]\left[\begin{array}{c} \alpha_{t}^{i,k}\\ \beta_{t}^{i,k} \end{array}\right]. \end{array}$$

For a new arriving frame, we can achieve *K* tracking results $\left\{{\hat{x}}_{t}^{i}|i=1,\ldots ,K\right\}$. For *i*th tracker, *z*_*t*_^*i*,*k*^ denotes the candidate image patch represented by *k*th features and *f*_*R*,*t*_^*k*^ **α**_*t*_^*i*,*k*^is the reconstructed appearance for *z*_*t*_^*i*,*k*^.

Then, a two-stage sparse-coded method by *ℓ*_2__RLS is used to obtain the coefficient vectors **α**_*t*_^*i*,*k*^ and **β**_*t*_^*i*,*k*^ as follows:

(11) α1=arg minαti,k,βti,k f1α1−zti,k2,s.t. αti,k2≤K1, βti,k2≤K2,

where *K*_1_ and *K*_2_ are nonzero components.

To effectively tackle the high-dimensional data in feature space, we use the diagonal matrix *W* to decrease the dimension of the feature space. For a set of samples *X* = {*x*_*t*_^*i*^ ∈ *ℝ* ^1×*p*^∣*i* = 1,…, *K*}, the joint sparse solution is shown as follows:

(12) α1,W=arg⁡minα1,W⁡ λWf1α1−Wzti,k22+γFW,X+τ1α122+τ2diag⁡W22,

where *F*(*W*, *X*) is the loss function and *τ*_1_, *τ*_2_ are the sparse parameters. If *W*_*ii*_ ≠ 0, the *i*th feature is activated.

The loss function is computed as

(13) $$\begin{array}{c} F\left(\;W,X\;\right)=e^{-\sum_{i=1}^{K}\left(x_{t}^{i}w_{t}^{i}\right)}, \end{array}$$

where {*w*_*t*_^*i*^ ∈ *ℝ* ^*p*×1^∣*i* = 1,…, *K*} is the sparse vector. If *w*_*i*_ ≠ 0, the *i*th feature is selected.

Then, the solution to the minimum loss function *F*(*W*, *X*) is achieved by solving the sparse problem as

(14) wti∗=arg minwti Xwti2,s.t. wti2≤K0,

where *K*_0_ denotes the maximum number of features that can be selected.

Considering the spatial neighborhood information of the image patch, let *N*_*w*_*t*_^*i*^_(*i*, *j*) denote the *j*th neighbor of *i*th feature; then the vector set is

(15) $$\begin{array}{c} z_{t}^{i}={\left(\;w_{t}^{i}\;\right)}^{2}+\overset{\tau }{\underset{j=1}{\sum }}\theta_{j}^{2}N_{w_{t}^{i}}^{2}\left(\;i,j\;\right),\;i=1,\ldots ,p, \end{array}$$

where *θ* is the weight of the neighbors.

The diagonal matrix *W* is formed as

(16) $$\begin{array}{c} {\left(\;W_{t}^{i}\;\right)}_{j,j}=\left\{\begin{array}{cc} 1, & {\left(w_{t}^{i}\right)}_{j}^{*}\neq 0\\ 0, & \text{otherwise}. \end{array}\right. \end{array}$$

From the above first-stage sparse representation coding, we take account of the spatial relationship of neighborhood features, which is beneficial for selecting a set of discriminative features to separate target from its background and reducing the computational burden by *ℓ*_2__RLS to solve the sparse approximation problem, as the target templates always contain some features from background, which is not the same as its neighbors. By doing discriminative feature selection as above, we can efficiently eliminate the features from background in the target templates. Therefore, we can construct a more efficient and robust target template library.

In the second sparse reconstruction stage, **α**_*t*_^*i*,*k*^ and **β**_*t*_^*i*,*k*^ in (12) can be computed as follows:

(17) αti,k,βti,k=arg minαti,k,βti,k Wf1α1−Wzti,k2,s.t. αti,k2≤K1, βti,k2≤K2.

The nonzero row of matrix *W* forms the matrix *W*′ ∈ *ℝ* ^*K*_0_×*p*^; let *f*_1_′ = *Wf*_1_, *z*_*t*_′ = *W*′*z*_*t*_, and *β*′ = *W*′**β**.

Then,

(18) αti,k∗,βti,k∗=arg minαti,k,βti,k f1′,W′αti,kβti,k−zt′2,s.t. α2≤K1, β2≤K2,

where *K*_1_, *K*_2_ are the sparsity parameters that control the sparse representation of the target template and the tolerance of interference in complicated environment, respectively.

Therefore, the reconstructed object appearance ${\overset{-}{f}}_{R,t}^{i,k}$ for *z*_*t*_^*i*,*k*^ is represented as

(19) $$\begin{array}{c} {\overset{-}{f}}_{R,t}^{i,k}=f_{R,t}^{i,k}\alpha_{t}^{i,k}. \end{array}$$

After above sparse reconstruction, the feature dimension reduced from *p* × *L* to *K*_0_ × *L* and *L* is the number of templates in the target library.

The predicted reliable object state for *i*th tracker at frame *t* is

(20) $$\begin{array}{c} {\hat{x}}_{t}^{i}=\underset{x_{t}}{\arg\max}\;p\left(\;x_{t}\;|\;z_{1:t},k_{t}^{i}\;\right). \end{array}$$

Then the corresponding tracking likelihood function of the *i*th tracker at frame *t* is

(21) $$\begin{array}{c} p\left(\;z_{t}\;|\;k_{t}^{i},z_{1:t-1}\;\right)=p\left(\;z_{t}\;|\;{\hat{x}}_{t}^{i}\;\right). \end{array}$$

In the proposed method, we use instantaneous and reconstructed features to describe the transient and stable appearance changes. The reliability of each tracker is

(22) pzt ∣ x^tipIzt ∣ x^tipRzt ∣ x^ti=∏k=1Kpzt ∣ x^ti,fI,tkpzt ∣ x^ti,fR,tk,

where $p_{I}\left(z_{t}|{\hat{x}}_{t}^{i}\right)$ is the instantaneous appearance likelihood based on the transient object appearance ${\overset{-}{f}}_{I,t}^{k}$, which is formed by a set of recent frames features *f*_*I*,*t*_^*k*^. $p_{R}\left(z_{t}|{\hat{x}}_{t}^{i}\right)$ is the reconstructed object appearance likelihood based on the reconstructed object appearance ${\overset{-}{f}}_{R,t}^{i,k}$ and ${\overset{-}{f}}_{R,t}^{i,k}$ comes from the stable object appearance, which is formed by *k*th feature and the tracking result *z*_*t*_^*i*,*k*^ from the *i*th tracker:

(23) pIzt ∣ x^ti,fR,ti,k=exp⁡−ρf−I,tk−zti,k2,pRzt ∣ x^ti,fR,ti,k=exp⁡−ρf−R,ti,k−zti,k2,

where *ρ* is the control parameter.

### 2.4. Predication and Update

To robustly track the target object, we update the tracker probability of the multiple trackers and the reliability of each tracker.

The tracker probability is updated as follows:

(24) $$\begin{array}{c} P\left\{\;k_{t}^{i}\;|\;z_{1:t}\;\right\}=\frac{p\left(z_{t}\;|\;k_{t}^{i},z_{1:t-1}\right)}{p\left(z_{t}\;|\;z_{1:t-1}\right)}P\left\{\;k_{t}^{i}\;|\;z_{1:t-1}\;\right\}, \end{array}$$

where

(25) Pkti ∣ z1:t−1=∑j=1KPkti ∣ kt−1j,z1:t−1Pkt−1j ∣ z1:t−1pzt ∣ z1:t−1=∑i=1Kpzt ∣ kti,z1:t−1·∑j=1KPkti ∣ kt−1j,z1:t−1Pkt−1j ∣ z1:t−1.

The corresponding observation model *p*(*z*_*t*_∣*x*_*t*_, *k*_*t*_^*i*^) for each tracker is updated based on the incremental subspace model in [4]. Then, the particle filter is used to approximate the state posterior distribution *p*(*x*_*t*_∣*z*_1:*t*_, *k*_*t*_^*i*^) by a set of *N* particles [24], the particles size *N* = 600:

(26) $$\begin{array}{c} p\left(\;x_{t}\;|\;z_{1:t},k_{t}^{i}\;\right)\approx \overset{N}{\underset{q=1}{\sum }}w_{q,t}^{i}\delta \left(\;x_{q,t}^{i}-x_{t}\;\right), \end{array}$$

where *δ*(·) is a delta function and {*w*_*q*,*t*_^*i*^}_*q*=1_^*N*^ is the sample weight associated with {*x*_*q*,*t*_^*i*^}_*q*=1,…,*N*_.

The particles *x*_*q*,*t*_^*i*^ are obtained from state prediction *p*(*x*_*t*_∣*x*_*t*−1_, *k*_*t*_^*i*^), which is simplified by first-order Markov model

(27) $$\begin{array}{c} x_{q,t}^{i}\sim p\left(\;x_{t}\;|\;x_{t-1},k_{t}^{i}\;\right). \end{array}$$

The weights are updated as

(28) $$\begin{array}{c} w_{q,t}^{i}=w_{q,t-1}^{i}p\left(\;z_{t}^{}\;|\;x_{t},k_{t}^{i}\;\right). \end{array}$$

Then, we achieve a set of *K* reliable states by maximizing the posterior estimates:

(29) x^ti=xq^,ti,q^=arg⁡maxq⁡wq,ti ∣ i=1,…,K,  q=1,…,N.

In the proposed method, the target appearance is constructed by multiple features that take account of transient and stable appearance changes to cope with occlusion and other interferences in complicated environments. For example, in a dynamic environment with drastic occlusion or illumination changes, the stable features are rarely updated, but the transient features can effectively describe the frequent appearance changes, while in a static background, if a background sample is added into the template, it usually has a good reconstruction with high likelihood because background is static at most of the time. Because the incorrect template is nonlinear, which is not the same as its neighbors, the two-stage sparse coding method taking account the spatial relationship of neighborhood features can prevent it from being selected. Therefore, we can construct a more efficient and robust target template library.

In addition, we update the template library based on the current tracking result as done in IVT method [4]; the samples with high likelihood and near the target are added to the template library. We repeat this procedure for each frame in the entire sequences. The tracking based on joint multiple feature representation and hierarchical sparse coding can provide a robust and accurate tracking result.

## 3. Tracking Based on Online Hierarchical Sparse Representation of Multifeature

As described above, the main step of the proposed tracking algorithm is shown in Algorithm 1.

**Algorithm 1 (tracking based on hierarchical sparse representation of multifeature).**

 

*Input*. There are the initial states of target {*x*_0_^*i*^ = *x*_0_∣*i* = 1,…, *K*}.

*Initializing*. Construct *L* training samples *X* ∈ *ℝ* ^*L*×*p*^, the set of samples for particle filter is {*x*_*q*,0_^*i*^, *w*_*q*,0_^*i*^ = 1/*N*∣*q* = 1,…, *N*}, and the tracker probability is {*P*_0_^*i*^{·} = 1/*K*∣*i* = 1,…, *K*}.

  For *t* = 1 to the end of video sequence, consider the following:
    For *i* = 1 : *K*,
  (1) achieve the solution to minimize loss function *F*(*W*, *X*) by (14),
  (2) construct diagonal matrix *W* by (16),
  (3) for candidate sample *z*_*i*_ in state *x*_*t*_^*i*^, achieve the sparse representation coefficients **α**_*t*_^*i*,*k*^ and **β**_*t*_^*i*,*k*^ by performing (18),
  (4) predict the reliable object state of each tracker at frame *t* by (20),
  (5) compute the state posterior distribution *p*(*x*_*t*_∣*z*_1:*t*_, *k*_*t*_^*i*^) by a set of *N* particles as (26),
  (6) predict state samples {*x*_*q*,*t*_^*i*^, *w*_*q*,*t*_^*i*^}_*q*=1_^*N*^ by (27),
  (7) update the sample weights *w*_*q*,*t*_^*i*^ as (28),
  (8) achieve a set of *K* reliable states ${\hat{x}}_{t}^{i}$ from the *i*th tracking by (29),
    End
  (9) compute the tracking likelihood function *p*(*z*_*t*_∣*k*_*t*_^*i*^, *z*_1:*t*−1_) of the *i*th tracker at frame *t* by (21),
  (10) update the tracker probability *P*{*k*_*t*_^*i*^∣*z*_1:*t*_} using (24),
  (11) the tracking result at the *t*th frame is achieved by (7),
  (12) update the training set and template library with the tracking results.
  End.

## 4. Experiments

To analyze the performance of the proposed tracking method, we compared our method with other five state-of-the-art trackers [25] such as IVT [4], L1 [6], MIL [5], OAB [26], and VTD [27] on several challenging video sequences. The target objects in the test videos are either nonrigid or rigid objects that suffered significant pose variation, heavy occlusion, in-plane and out of plane rotation, or motion blur. The video sequences are available in https://sites.google.com/site/trackerbenchmark/benchmarks/v10. The proposed tracker algorithm is implemented in MATLAB, which is run on a PC with 2CPU, 2.5 GHz, and 3.1 GB RAM, at around 20 frames per second.

### 4.1. Parameters Setting

For all test video sequences, we manually select the initial target location. Each image patch is normalized to 32*∗*32 pixels and sparsity parameters *τ*_1_ = *τ*_2_ = 0.001 and *γ* = 0.1 and the dimensions of intensity features, HOG features, and Haar-like feature is 1024, 1296, and 1760, respectively. The number of particles is *N* = 600, and the number of template samples is *L* = 16.  Table 1 lists the characteristics of the evaluated sequences used in the experiments of this paper.

### 4.2. Qualitative Comparison

**Experiment 1 (illumination variation, occlusion, scale change, and fast motion of rigid object).**

**Figure 2:**
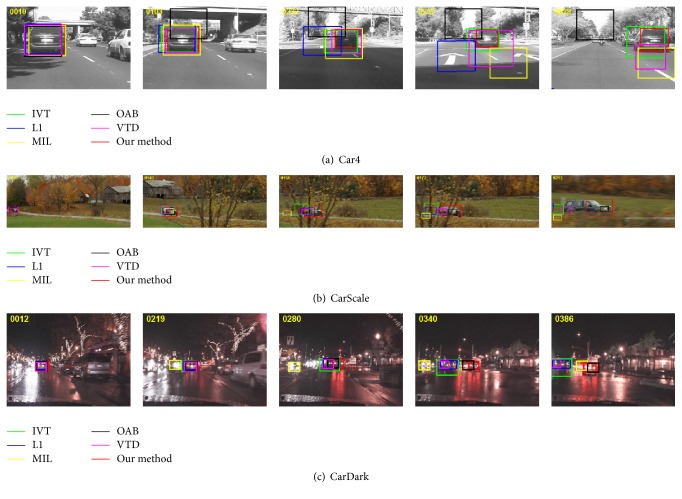
The tracking results of the rigid object undergoing severe occlusion, illumination, and scale change.

The sequence of Car4 is to track a car in an open road with illumination variation and partial occlusion as shown in Figure 2(a). At frame 86, the OAB tracker appears to slightly drift due to the trees and bridge occlusion and fails to track the car at frame 233. The L1, MIL, and VTD trackers start to drift away from the target when drastic illumination changes occur at frame 195 and fail to track the target at frame 255. The IVT and the proposed method can successfully track the target because they dynamically updated the template, which is beneficial for coping with the occlusion and illumination changes. However, the result of IVT is less satisfied because the tracking box is larger than the target object from frame 195 to the end sequences.

In the CarScale sequence, the tracking target is a fast motion car in an open road. Compared with the Car4 sequence, this sequence is more challenging because the tracked car undergoes large scale changes and fast motion on the entire sequence. Due to the fast motion accompanied with the tree's occlusion, IVT, L1, MIL, and VTD trackers drift with different degree at frame 164 and finally lost the target at frame 171. The proposed method gives the best results followed by the OAB tracker.

The CarDark sequence is challenging because the target object undergoes the motion blur in a night environment with low contrast and strong reflection interference. Due to the strong reflection interference, the MIL tracker drifts a little from the target at frame 122 and lost the target at frame 202 and then regards the other car as the tracked target. The IVT, L1, and VTD trackers drift away from the target at frame 277. The OAB tracker performs well on this sequence and yields the second best results. The proposed method can accurately track the target object in the whole sequence with small center position error and high overlap rate.

**Experiment 2 (occlusion, scale change, and rotation of nonrigid object).**

**Figure 3:**
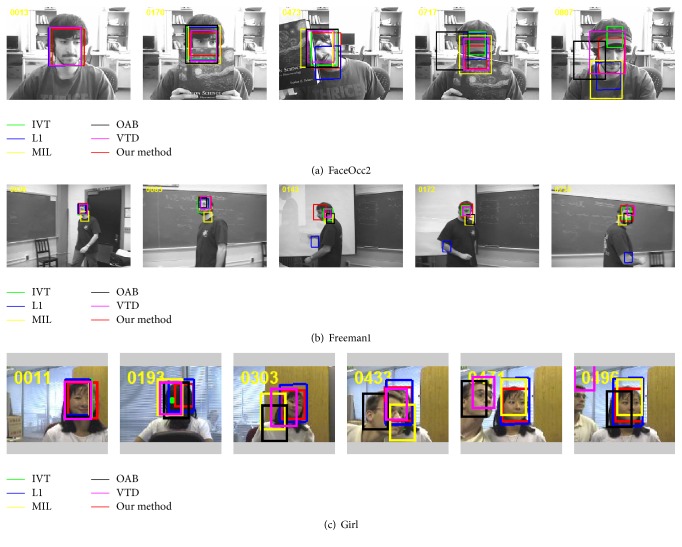
The tracking results of object undergoing occlusion, scale change, and rotation.

The target object in FaceOcc2 sequence undergoes the drastic occlusion and in-plane rotation. As shown in Figure 3(a), when there is a small occlusion with a book at frames 128~185 and frames 245~279, all methods perform well. But when partial occlusion and in-plane rotation occur together at frames 392~510, most of trackers have poor performances. When the target almost fully occludes by a book and a hat at frames 688~740, all methods except this paper method drift away from the target at different degrees. Since the proposed method uses multiple complementary features to build transient and stable appearance models and update the template library online, it can effectively handle the occlusion and give satisfactory tracking results.

The Freeman1 sequence is challenging because the interested man's face undergoes large scale changes and view variations. Due to the large scale changes, MIL drifts away from the target at frame 32. As the view changes from the left to right, the L1 and OAB trackers totally lost the target at frames 131 and 176, respectively. The tracking methods like IVT, VTD, and the proposed method perform well on this challenging sequence and can track the target accurately.

The Girl sequence has drastic appearance changes because of the out of plane rotation and similar target occlusion. When out of plane rotation occurs at frames 90~122 and 169~260, IVT tracker totally fails to track the Girl's face; other trackers drift at different degrees. The OAB and VTD trackers fail to track the target object and track the similar target when the Girl's face is occluded by a man's face at frames 420~470. MIL tracker can successfully track the target except some errors like frames 303 and 433. L1 tracker and the proposed method perform well on this sequence.

**Experiment 3 (illumination, scale change, and occlusion of nonrigid object).**

The track target in shaking sequence undergoes drastic illumination and poses changes on the whole video sequence. It brings more challenges to accurately track the target because the color of object appearance is similar to the stage lighting. IVT and OAB almost fail to track the target at frame 23 and cannot recover at the rest frames. The MIL tracker drifts a little at frame 61 due to the drastic illumination changes. Although the stage lights change drastically accompanied with the serious head shaking, the L1 and VTD trackers perform well except some errors. The proposed method can effectively adapt to the severe object appearance changes when those variations occur together and achieve satisfactory results.

**Figure 4:**
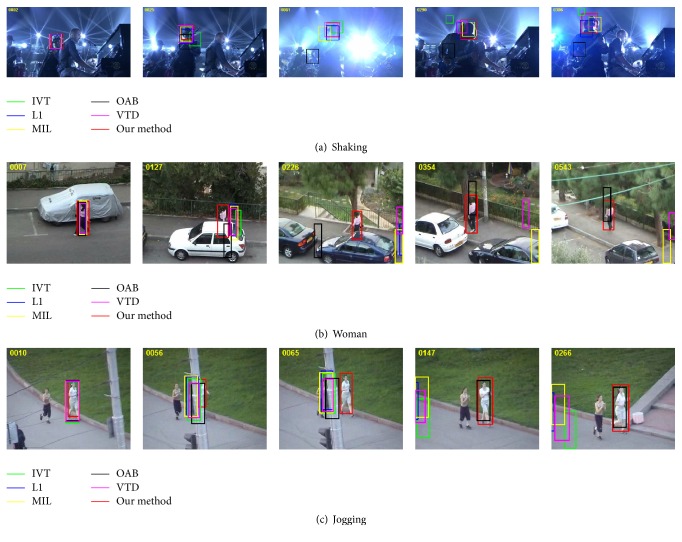
The tracking results of object undergoing severe occlusion, illumination, and pose changes.

The Woman sequence is very challenging because the target object undergoes large scale changes, view variations, and occlusions simultaneously. As shown in Figure 4(b), all trackers merely perform well except the proposed method. The results of the proposed method show slight drift; other methods like IVT, L1, MIL, OAB, and VTD trackers totally lose the target when heavy occlusion occurs at frame 130 and never recover to track the target in the subsequent video sequence except the OAB tracker. The OAB tracker recaptures the target at frame 337 and keeps to track the target until the end of sequence with a little drift.

The Jogging sequence is more challenging to track because the tracked target is fully occluded by a stem and undergoes large scale change and fast motion simultaneously. The IVT, L1, MIL, OAB, and VTD trackers completely fail to track the target when the target is fully occluded by a stem at frames 50~62, and the OAB tracker recaptures the target at frame 106. The proposed method can accurately track the target on the entire sequence.

From some sampled tracking results of the proposed method and other five methods on 9 image sequences, we can conclude that the algorithm in this paper can accurately and robustly track the target under the environment with illumination variation, scale change, and motion blur.

### 4.3. Quantitative Comparison

Two metrics are used to evaluate the proposed tracker with reference trackers in gray-scale videos. The first is the center position error, which is applied to evaluate the distance between the ground-truth center *R*_*g*_ and tracked object center *R*_*t*_ (in pixels) at each frame by the Euclidean distance. The other metric is the overlap rate [28], which is defined as score = area(*R*_*t*_∩*R*_*g*_)/area(*R*_*t*_ ∪ *R*_*g*_), where *R*_*t*_ denotes the bounding box generated by a tracking method and *R*_*g*_ is the ground-truth bounding box.

**Figure 5:**
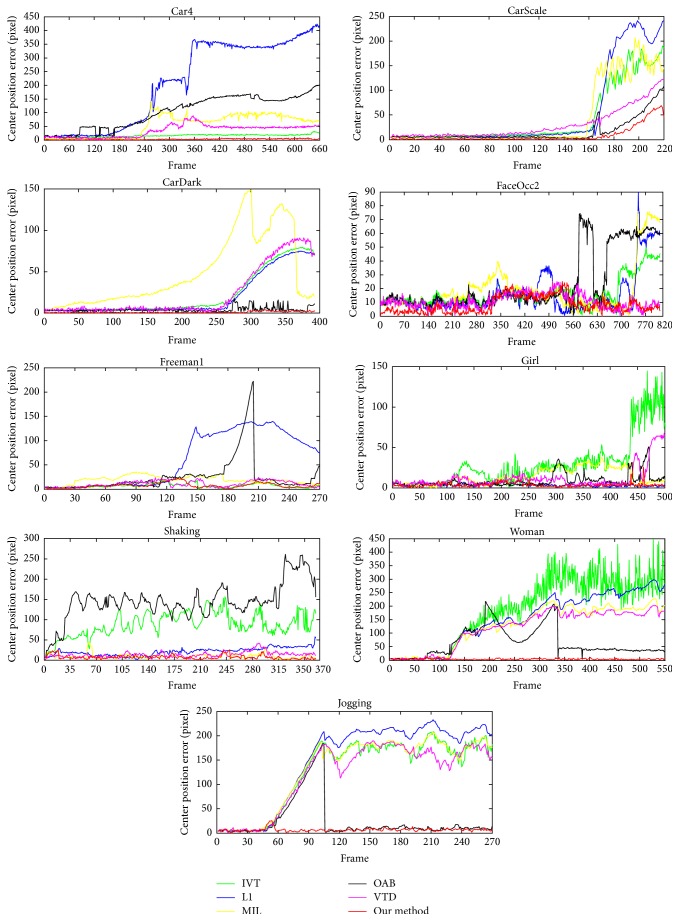
The center position errors of different trackers on 9 video sequences.

**Figure 6:**
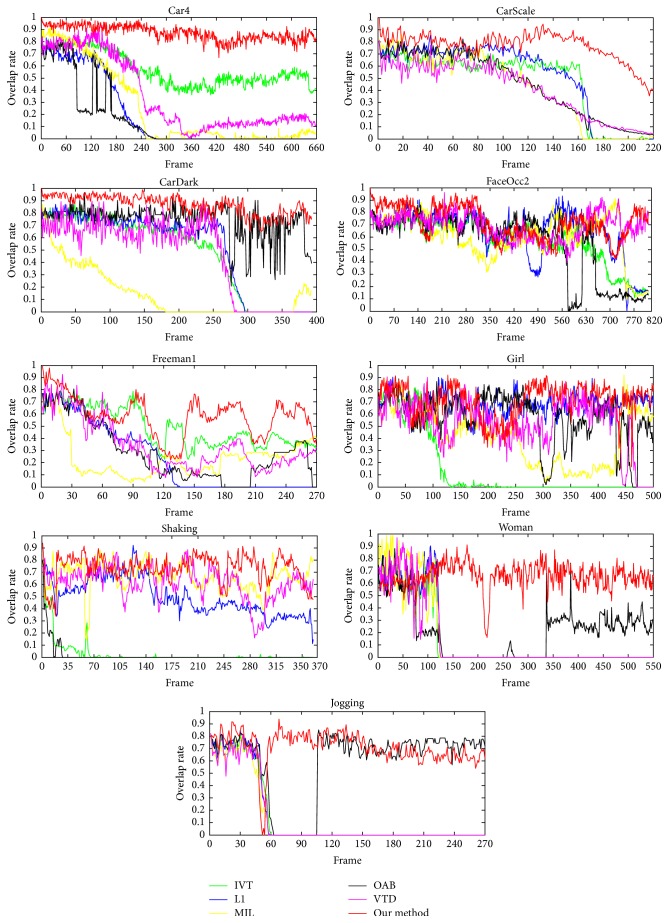
The overlap rate evaluation of different trackers on 9 video sequences in each frame.

Table 2 and Table 3 show the average center position errors and the average overlap rate for all trackers. Figures 5 and 6 show the center position error curve and overlap rate evaluation curve of different trackers on 9 video sequences at each frame. It can be seen that the proposed algorithm has the optimal or suboptimal performance in terms of average center position errors and average overlap rate in most test video sequences compared with other methods. Most competing tracking methods do not give a satisfactory result; the center position error is larger and the overlap rate is lower. The average position error of this paper at 9 videos is only 5.53 pixels, which is far less than other trackers; the average central position errors of other trackers like IVT, L1, MIL, OAB, and VTD trackers are 59.58 pixels, 76.14 pixels, 51.91 pixels, 46.01 pixels, and 41.54 pixels, respectively. The average overlap rate of the proposed method reaches 73.6%, which is higher than other trackers; the average overlap rates of IVT, L1, MIL, OAB, and VTD trackers are 39.9%, 34%, 40%, 33.7%, and 32.7%, respectively. They both highlight the advantages of the algorithm in this paper. Overall, the effectiveness of the proposed tracker method is verified.

## 5. Conclusion

In this paper, we propose a robust tracking algorithm that leverages hierarchical sparse coding to optimize the image representation from multifeature. We compare our tracking method with other five state-of-the-art trackers on nine sequences to validate the robustness and accurateness of the proposed method. The experiment results show that our method can effectively and robustly handle the challenging scenes where the target object undergoes drastic variation in pose, scale, rotation, occlusion, and illumination. The success of our method can be attributed to constructing multiple observation models that form the multifeature by hierarchical sparse coding, which takes the spatial relationship of neighborhood features into consideration and solves the sparse approximation problem by *ℓ*_RLS. The appearance model constructed by instantaneous and stable appearance features with two-stage sparse representation coding is more robust to cope with appearance change in complex environment and more effective to select a set of discriminative features to separate the target from its background. In the proposed method, we compute the reliability of each tracker by the tracker likelihood function that accounts for transient and reconstructed appearance model and select the most reliable one among multiple trackers. The training set and the template library are both incrementally online updated. All of these are beneficial to cope with the appearance change and can improve the tracking performance in dynamic environments.

However, the limitation of the proposed method is mainly focused on the following. (1) The tracking system is not effective enough for real-time tracking because multifeatures are calculated at the same time for test video sequences, which is time-consuming. In addition, it cannot be adapted to extract the feature according to the video attribute. (2) The ability of each feature to describe the target cannot be effectively measured. (3) It cannot successfully track the target when the object leaves out the scene but reappears in subsequent frames.

In the future, we will improve the proposed method in some aspects. (1) We will improve the algorithm in real-time by proposing a method to adaptively extract the multifeatures according to the video attribute, which can reduce the time and computation load of the feature extraction. (2) We would improve the tracking performance by introducing the occlusion mechanism and drift mechanism, which can alleviate updating the template with wrong samples when the target object is occluded or drifted. Both strategies are useful to deal with appearance changes and beneficial to robustly track the target in complex environments.

## Figures and Tables

**Table 1:** Tracking sequence in the experiments.

Sequence	Frame	Main challenge
(1) Car4	659	Occlusion and illumination change
(2) CarScale	252	Occlusion and scale change
(3) CarDark	393	Illumination change, occlusion, and motion blur
(4) FaceOcc2	812	Occlusion and in-plane rotation
(5) Freeman1	326	Scale change and view change
(6) Girl	500	Out of plane rotation, occlusion, and scale and pose change
(7) Shaking	365	Illumination change and pose change
(8) Woman	569	Scale change, view variation, and occlusion
(9) Jogging	307	Occlusion, abrupt motion, and scale change

**Table 2:** The average position errors (pixels).

Video	IVT	L1	MIL	OAB	VTD	Our method
Car4	*16.31*	209.22	55.10	103.49	34.46	**2.97**
CarScale	42.64	53.52	43.63	*15.66*	29.35	**8.44**
CarDark	22.03	19.85	48.41	*3.90*	23.29	**1.92**
FaceOcc2	16.21	15.54	18.17	24.32	*10.69*	**8.42**
Freeman1	**6.85**	61.74	18.98	25.44	10.71	*7.44*
Girl	29.37	*3.76*	14.11	8.55	11.70	**3.63**
Shaking	87.90	21.05	*9.59*	144.66	13.42	**7.11**
Woman	186.70	151.79	128.08	*63.76*	119.01	**3.27**
Jogging	128.29	148.81	131.14	*24.29*	121.31	**6.54**

*Average*	59.58	76.14	51.91	46.01	*41.54*	**5.53**

*Note*: the optimal result is shown as bold and the suboptimal one as italic.

**Table 3:** The average overlap rate for all of trackers.

Video	IVT	L1	MIL	OAB	VTD	Our method
Car4	*0.570*	0.196	0.257	0.151	0.357	**0.873**
CarScale	0.469	*0.515*	0.421	0.451	0.408	**0.768**
CarDark	0.480	0.513	0.153	*0.741*	0.461	**0.868**
FaceOcc2	0.572	0.641	0.611	0.549	*0.70*	**0.723**
Freeman1	*0.493*	0.234	0.241	0.288	0.343	**0.587**
Girl	0.147	*0.676*	0.404	0.577	0.521	**0.710**
Shaking	0.032	0.50	*0.663*	0.017	0.613	**0.735**
Woman	0.129	0.145	0.147	*0.217*	0.144	**0.654**
Jogging	0.139	0.142	0.127	*0.60*	0.134	**0.711**

*Average*	0.337	0.396	0.336	0.399	*0.409*	**0.736**

*Note*: the optimal result is shown as bold and the suboptimal one as italic.
